# Automated, High‐Throughput Phenotypic Screening and Analysis Platform to Study Pre‐ and Post‐Implantation Morphogenesis in Stem Cell‐Derived Embryo‐Like Structures

**DOI:** 10.1002/advs.202304987

**Published:** 2023-11-22

**Authors:** Vinidhra Shankar, Clemens van Blitterswijk, Erik Vrij, Stefan Giselbrecht

**Affiliations:** ^1^ MERLN Institute for Technology‐Inspired Regenerative Medicine Department for Instructive Biomaterials Engineering (IBE) Maastricht University Maastricht 6229ET The Netherlands

**Keywords:** bioengineering platform, high‐content imaging, microwells, screening, stem cell‐based embryo models

## Abstract

Combining high‐throughput generation and high‐content imaging of embryo models will enable large‐scale screening assays in the fields of (embryo) toxicity, drug development, embryogenesis, and reproductive medicine. This study shows the continuous culture and in situ (i.e., in microwell) imaging‐based readout of a 3D stem cell‐based model of peri‐implantation epiblast (Epi)/extraembryonic endoderm (XEn) development with an expanded pro‐amniotic cavity (PAC) (E3.5 E5.5), namely XEn/EPiCs. Automated image analysis and supervised machine learning permit the identification of embryonic morphogenesis, tissue compartmentalization, cell differentiation, and consecutive classification. Screens with signaling pathway modulators at different time windows provide spatiotemporal information on their phenotypic effect on developmental processes leading to the formation of XEn/EPiCs. Exposure of the biological model in the microwell platform to pathway modulators at two time windows, namely 0–72 h and 48–120 h, show that Wnt and Fgf/MAPK pathway modulators affect Epi differentiation and its polarization, while modulation of BMP and Tgfβ/Nodal pathway affects XEn specification and epithelialization. Further, their collective role is identified in the timing of the formation and expansion of PAC. The newly developed, scalable culture and analysis platform, thereby, provides a unique opportunity to quantitatively and systematically study effects of pathway modulators on early embryonic development.

## Graphical abstract

This article describes a novel and scalable microwell platform for performing high‐throughput screens of stem cell‐derived embryo‐like structures. The modular analysis pipeline uses discrete classifiers to phenotypically quantify the structures. Screening of signaling pathway modulators demonstrate that the formation and patterning of embryonic morphogenesis, including pro‐amniotic cavity (PAC) formation, is spatiotemporally controlled by Wnt, Fgf/MAPK, BMP, and Nodal pathways.

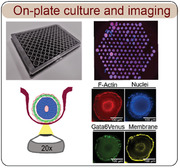

## 1 Introduction

Early mammalian embryonic development is characterized by crucial cell fate decisions of the embryonic and extra‐embryonic compartments that ensure developmental progression.^[^ ^1^ ^]^ The recent advent of stem cell‐based embryo models creates new exciting opportunities to study pre‐ and early post‐implantation embryo differentiation and morphogenesis. Typically, embryo models, including blastoids,^[^ ^2^ ^]^ extraembryonic/embryonic peri‐implantation models,^[^ ^3^ ^]^ and gastruloids,^[^ ^4^ ^]^ are formed by coaxing stem cells within a non‐adhesive (micro‐) environment that supports their unrestricted self‐organization potential.^[^ ^2^, ^5^ ^]^ Embryo models are capable of recapitulating sequences of developmental processes,^[^ ^6^ ^]^ modeling developmental defects,^[^ ^6^, ^7^ ^]^ and providing insights into the molecular regulators driving development.^[^ ^2^, ^8^ ^]^ Moreover, they have the potential to reveal hidden complexities in embryo morphogenesis and the assemblage of cell lineages at various stages of development.

Despite their utility, embryo models exhibit significant morphological heterogeneity, which hinders inferring their relevance to the study of natural embryo development. Moreover, the lack of automated tools to quantify embryonic morphogenesis and associated stages in development limits their potential applications in the screening domain. Part of these challenges may be addressed by forming large numbers of embryo models to determine true variability, as has been done in organoid models.^[^ ^9^ ^]^ Formulating a better understanding of how different tissues coordinate intricate morphogenetic events; studying the impact of mechanobiological cues, genetic determinants, and drugs; and performing toxicological studies on a large scale would be important in navigating the applications of such embryo models.

Around embryonic day (E) 3.25 to E4.0, the embryonic compartment in the blastocyst establishes lineage segregation based on a Nanog‐Gata6 bias^[^ ^10^ ^]^ thereby causing a bifurcation into epiblast (Epi) and primitive endoderm (PrE) regulated by the fibroblast growth factor (FGF)/extracellular signal‐regulated protein kinase (ERK) pathway.^[^ ^11^ ^]^ From E4.0 to E5.0, the PrE spatiotemporally patterns into an epithelialized layer enveloping the Epi, a process regulated by the bone morphogenetic protein (BMP) pathway,^[^ ^12^ ^]^ polarizing factors such as aPKC and DAB2^[^ ^13^ ^]^ and supported by cell‐surface fluctuations.^[^ ^6^, ^14^ ^]^ Simultaneously, the Epi undergoes a rosette‐like transition with an apical‐basal polarity guided by the wingless‐related integration site (WNT) pathway, activin signaling, and mitogen‐activated protein kinase (MEK)/ERK pathway.^[^ ^15^ ^]^ The polarization of Epi triggers the formation of a pro‐amniotic cavity (PAC), which is facilitated by the reinforcement of E‐cadherin and negatively charged sialomucins causing a repulsive force, thereby allowing a water influx into the forming lumen.^[^ ^16^ ^]^ The whole sequence of events is well‐coordinated in space and time, making it challenging to decouple the effect of individual pathways on these morphogenetic events.

Technological advances in micro‐engineering platforms and image‐based analysis software have enabled researchers to realize some potential applications of clinical and pharmaceutical research using embryo models.^[^ ^5^, ^17^ ^]^ 3D high‐content phenotypic screening requires a versatile platform to allow long‐term culture, imaging, and quantification; robust culture conditions to allow reproducibility; simple and efficient read‐out, and supervised analysis to extract the most reliable information.^[^ ^18^ ^]^ Soluble factors or compounds’ screening can serve as a powerful tool to study the function of signaling pathways in development.^[^ ^9^, ^18^, ^19^ ^]^ Supervised machine learning^[^ ^18b^ ^]^ and deep learning^[^ ^18c^ ^]^ tools can be of key value for analyzing complex image‐based datasets of in vitro models of development. Especially in the field of in vitro embryo research, they enable systematic filtering of the feature/phenotype of interest. However, there is a need for more accessible, free, user‐friendly systems to perform such large‐scale automated analysis on in vitro stem cell‐derived 3D models.

Here, we show the continuous culture and in situ imaging‐based readout of a 3D stem cell‐based model of pre‐ to early post‐implantation development of the mouse embryo. We use our previously developed model, named XEn/EPiCs, where we induce the co‐development of the extraembryonic endoderm (XEn) and the epiblast (Epi) tissue of the pre‐implantation blastocyst embryo.^[^ ^3c^ ^]^ We show the highly efficient formation of XEn/EPiCs within a polymer film‐based thermoformed microwell platform^[^ ^20^ ^]^ that is, because of its thin and transparent walls, compatible with high‐throughput and high‐content screening. In these microwells, XEn/EPiCs autonomously progress in development to the early post‐implantation stage, which is characterized by extraembryonic visceral endoderm‐like differentiation, deposition of a lamina‐rich basement membrane onto the polarizing Epi, and the formation of PAC. This sequence recapitulates XEn and Epi formation and morphogenesis of the E4.0 – E5.5 window of mouse embryo development.^[^ ^3c^ ^]^ We then perform screens using soluble factors over time to identify the signaling pathways underlying embryo differentiation and morphogenesis.

We use the open‐source image analysis software CellProfiler (CP)^[^ ^21^ ^]^ to analyze the large image data sets in an automated set‐up; identifying objects and quantifying parameters such as object size, shape, area, texture, intensity, and intensity distribution. Following this, we performed supervised machine learning using CellProfiler Analyst (CPA)^[^ ^22^ ^]^ to provide information about the occurrence of different phenotypic variants of XEn/EPiCs, the sizes of the structures and each compartment. The performance of automated quantification was comparable to manual scoring in terms of reproducibility and sensitivity to developmental perturbation by soluble signaling pathway modulators. The effect of different pathways on epiblast polarization, extraembryonic endoderm epithelialization, and lumenogenesis were correlated by conducting a morphogenetic screen with a small library of pathway modulators. We performed a primary screen with 40 modulators that activate or inhibit a specific pathway for the entire period of development. The modulators that showed a significant effect on the yield and morphology of XEn/EPiCs compared to the control were shortlisted for a secondary screen to decouple their effect at different time windows of exposure. We found that the time of exposure of XEn/EPiCs to some pathway modulators affected the size and occurrence of PAC formation. We show that some of the key pathways described in the literature to play a role during implantation, namely, Wnt,^[^ ^23^ ^]^ Fgf/MAPK,^[^ ^24^ ^]^ BMP/Tgfβ,^[^ ^12^, ^25^ ^]^ and activin/Nodal,^[^ ^26^ ^]^ also play a crucial role in the timely formation of the PAC. In summary, we show the application of a highly scalable and reproducible platform for performing high‐throughput screens and quantifying relevant tissue‐level data using a simple and modular pipeline.

## 2 Results

### 2.1 Generation and Characterization of XEn/EPiCs within Thermoformed Microwell Arrays to Study Pre‐ to Post‐Implantation Development

**Figure 1:**
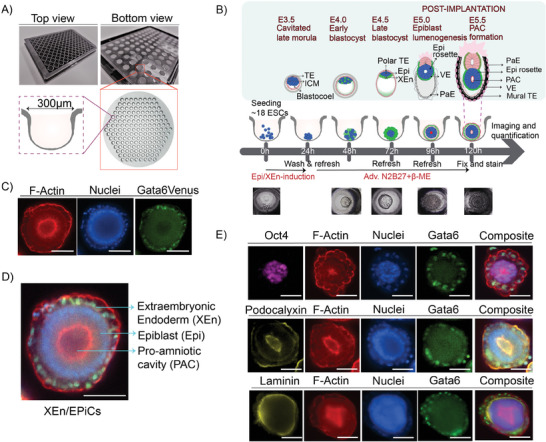
Generation and Characterization of Chemically‐Induced XEn/EPiCs: A) Thermoformed microwell screening plate with a zoomed‐in inset of a single microwell, B) Schematic depicting the formation of XEn/EPiCs within thermoformed microwells with a top panel indicating the developmental stage and features of a natural embryo they mimic, (ICM: Inner Cell Mass, TE: Trophectoderm, XEn: Extraembryonic Endoderm, Epi: Epiblast, VE: Visceral Endoderm, PaE: Parietal Endoderm, PAC: Pro‐Amniotic Cavity) and the bottom panel showing Epi/XEn induction: Chir, Fgf4, Retinoic acid, cAMP, Heparin, 5% FBS, C) Immunochemistry image of a XEn/EPiC stained for nuclei (Hoechst) and F‐actin (Phalloidin), D) A merged multichannel acquisition image of XEn/EpiCs, E) Immunochemistry characterization of XEn/EPiCs for Oct4, a pluripotency marker, Podocalyxin, a marker for the pro‐amniotic cavity lining and XEn lining, and Laminin, lining the epiblast. Scale bars: 100 µm.

A novel in situ imaging and analysis set‐up was created using thermoformed microwell screening arrays (Statarrays, 300MICRONS) which allowed the generation of XEn/EPiCs in large numbers and with sufficient control over cell numbers. The first step (**Figure** **1**A) involved, seeding an average of 18 mouse embryonic stem (ES) cells per microwell in a previously reported induction medium^[^ ^3c^ ^]^ consisting of Chir99021 (Chir), Retinoic Acid (RA), fibroblast growth factor 4 (Fgf4) with heparin, and 8Br‐cAMP (Figure 1B). The induction medium triggered the co‐development of both the embryonic Epi compartment and the enveloping XEn layer.^[^ ^3c^ ^]^ We chose an ES cell line containing a fluorescent reporter for the gene Gata6 (Gata6:H2B‐Venus) to visualize the formation of the extraembryonic endoderm (XEn). The ES cells were cultured in basic serum‐free medium containing advanced N2B27, which was daily refreshed. After 120 h of culture, in ≈75‐80% of microwells, ES cells had self‐organized into 3D, spherical structures consisting of an epithelialized extraembryonic endoderm (XEn) layer encasing a polarized epiblast‐like compartment (Epi) with a pro‐amniotic‐like cavity (PAC), namely XEn/EPiCs (Figure 1C,D). These structures could be directly imaged within the microwells and resemble the Epi and XEn compartments of an E5.5 stage embryo (Figure 1B).^[^ ^3^, ^27^ ^]^ The structures were further characterized for the expression of podocalyxin, which lines the PAC^[^ ^16a^ ^]^; Oct4, which marks the pluripotent epiblast^[^ ^28^ ^]^; and Laminin, which defines the basement membrane that is deposited by the XEn^[^ ^29^ ^]^ (**Table** **1** and Figure 1E). The addition of 5% of fetal bovine serum to the induction cocktail from 0–24 h improved the yield of XEn/EPiCs to 81% (Figure S5, Supporting Information).

**Table 1:** List of primary and secondary antibodies.

Antibody	Company	Dilution
Rabbit Anti‐Laminin	Sigma Aldrich L9393	1:200
Goat Anti‐Podocalyxin	R&D Systems MAB1556‐SP	1:300
Goat Anti‐Oct4	R&D Systems AF1759‐SP	1:100
Hoechst 33 342 (nuclei stain)	Invitrogen H3570	1:300
Phalloidin AF647 (F‐Actin stain)	Invitrogen 10 656 353 (Alexa Fluor 647)	1:300
WGA (Wheat Germ Agglutinin) (Cell membrane stain)	VWR 29077‐1 (CF568)	1:300

### 2.2 Imaging and Phenotypic Classification of XEn/EPiC Variants within Microwells

**Figure 2:**
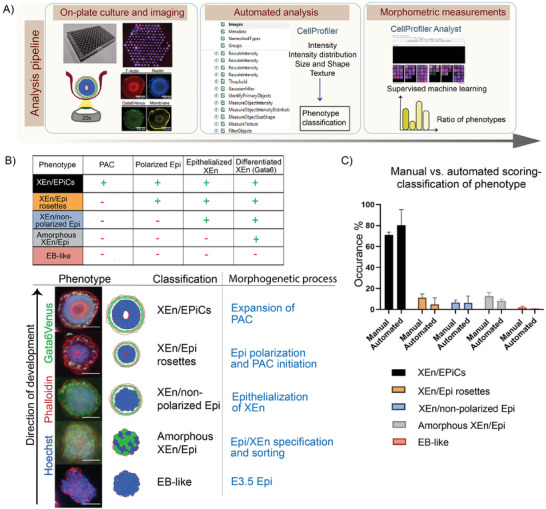
Imaging and phenotypic classification of XEn/EPiC variants within microwells: A) Schematic for the sequence of microwell array‐based culture, in‐microwell staining, fluorescence imaging, and automated morphometric feature extraction of XEn/EPiC variants. B) Nomenclature and classification of different morphologies observed and their features have (table). C) Percentage of manual versus automated measurement of the different XEn/EPiC variants. Scale bars: 100 µm.

In vitro cultures of organoids and stem cell‐based embryo models are associated with phenotypic heterogeneity,^[^ ^18b^ ^]^ reflecting variable stages in development or in vitro artifacts. Accordingly, aside from the XEN/EpiCs that we aimed to form, we observed the formation of multiple other phenotypes reflecting earlier stages along the developmental timeline (**Figure** **2**B). To classify these phenotypes, we imaged all embryo‐like structures within the microwells and analyzed them using the open‐source software package CellProfiler (CP). First, embryo‐like structures were fixed within the microwells at 120 h of culture and stained with Hoechst (nuclei), Phalloidin (F‐actin), and WGA (cell membrane). In addition, the Gata6:H2B‐Venus fluorescent expression depicted the XEn layer. Montage images containing all structures within the entire wells were acquired using a fluorescence spinning disk microscope (Nikon Ti‐E spinning disk; Figure 2A). Second, a CP pipeline was generated to identify all embryo‐like structures and extract their phenotypic features, including area, texture, size, shape, intensity, and radial intensity distribution, among others (e.g., Zernicke features, refer to CP manual for details on this feature^[^ ^30^ ^]^) (Figure 2A; Figure S1, Supporting Information). These phenotypic features can be used to pinpoint the morphogenetic processes occurring during the E3.5 – E5.5 window of mouse embryo development, and thus in which phenotypic class to place the embryo‐like structures. The morphogenetic processes that we used to specify these classes include XEn (i.e., primitive endoderm) differentiation (Gata6 expression), sorting of XEn cells toward the surface, the formation of a continuous XEn layer engulfing the Epi (positioning of Gata6+ cells), initiation of Epi polarization (F‐actin‐rich center within Epi), and formation of the PAC (cavity within the Epi).

The imaging within microwells at a 20x objective did not provide a very sharp image with single‐cell resolution. However, this objective was carefully chosen to detect and analyze the different morphologies and tissue compartments while achieving high‐throughput. The CP was able to perform image‐based quantitative analysis efficiently by utilizing the “Threshold” module to the detect F‐Actin and Hoechst channels and further allow the detection of the objects. On the one hand, since the goal was to perform morphometric measurements for the specific tissue compartment as a whole, single cell resolution of the cells was not the prerequisite. On the other hand, if the aim is to measure cellular features, single‐cell resolution can be obtained with a higher resolution using the 40x air‐immersion objective, which might increase the imaging time significantly. The quality can also be further improved by flushing the structures from the microwells (by gentle pipette mixing), transferring them to a glass‐bottom dish, and imaging them with an oil‐immersion 40x objective. This method can also be used to generate volumetric data of the whole 3D structure with a Z‐stack function and 3D reconstruction.

To obtain an automated classification process for the different phenotypes observed, the object measurements from CP were used to train a classifier – a set of rules for each phenotypic class – in CellProfiler Analyst (CPA), an open‐source CP extension for supervised machined learning. The classification included five different phenotypic classes, XEn/EPiCs (structures with an epithelialized XEn expressing Gata6, polarized Epi, and expanded PAC), XEn/Epi rosettes (structures with an epithelialized XEn expressing Gata6, polarized Epi, and an F‐Actin‐rich region on the apical side), XEn/Non‐polarized Epi (structures with an epithelialized XEn expressing Gata6 and non‐polarized Epi), EB‐like (ES cell aggregates without XEn specification and negative for Gata6 expression) and amorphous XEn/Epi (structures with disorganized Gata6 expressing XEn) (Figure 2B). The XEn/non‐polarized Epi, XEn/Epi rosettes, and XEn/EPiCs chronologically resemble the XEn and Epi compartment of the E4.0, E4.5, and E5.5 embryo, respectively, along the peri‐implantation developmental timeline (Figure 1B).

We used a supervised machine‐learning approach to find the linear combinations of phenotypic features that describe each of these phenotypic classes. The object scores that were generated for the automated object classification by CPA were validated by manual scoring, which showed a comparable yield of the different phenotypes (Figure 2C). Overall, we show the creation of a microwell‐based, high‐throughput culture and imaging pipeline to automatically detect and quantify the morphological diversity of XEn/EPiCs embryo models including developmental steps toward the E5.5 stage. We also observe a sufficient overlap between manual and automated measurements, making it a suitable tool to perform high‐content analysis of XEn/EPiCs. Quantification of the performance matrices for this supervised machine‐learning algorithm by the Random Forest method provided the accuracy, precision, recall and F1 scores for the training set, which were, 83.45%, 82.0%, 75.0%, and 78.0% respectively for XEn/EPiCs. (Figures S2 and S3, Supporting Information).

The performance scores could be further improved by training larger data sets. However, there is always a risk of increasing the confusion of the algorithm because the different phenotypes represent a sequential developmental stage and the difference between the different types varies by very close margins, for which an error in the consistency of supervised labeling may have been introduced as well. Additionally, by improving the imaging quality and by using tissue‐specific dyes, both the manual classification for supervised machine learning and the resulting algorithm could be made more robust. However, this improvement could have an impact on the imaging time and hence the throughput of the platform.

Examples of misclassifications (population within false positive and false negative) could occur due to larger sizes of some XEn/EPiCs in the periphery of the microwells as a result of the larger proportion of cells seeded there. This size variation could have caused a higher intensity of fluorophores, thereby confusing the sorting to bins. To overcome these misclassifications, it could help to filter out the peripheral microwells before performing the training. Another type of misclassification could happen between Epi rosettes and XEn/non‐polarized Epi because they mostly differ in their accumulation of the F‐actin bundle (only present in the former). This misclassification could be overcome by improving the training sets and supervised classification for these 2 phenotypes with better bonafide images representing these stages.

### 2.3 High‐Content Screening of XEn/EPiC Variants with Signaling Pathway Modulators

**Figure 3:**
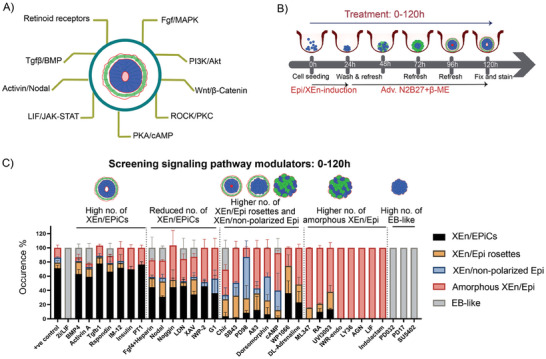
High‐content phenotypic screening of XEn/EPiCs with signaling pathway modulators from 0–120 h of development: A) Signaling pathways targeted by the library with signaling compounds, B) Schematic of the formation and treatment of XEn/EPiCs with compounds, C) Graphs showing the occurrence of different XEn/EPiC variants observed upon exposure to compounds. The compounds were classified based on the dominant differential phenotypes observed compared to the control (base media + 0.1% DMSO from 0–120 h); Data are mean ± s.d; *n* = 3 replicates; in each replicate ≈150 structures were analyzed.

To identify essential signaling pathways during pre‐ to early post‐implantation development, we performed high‐content screening using a custom library of signaling pathway modulators that are known to play an important role in this window of embryo development. This library contains modulators targeting the following pathways: Wnt, Fgf/MAPK, Tgfβ/BMP, activin/Nodal, ROCK, PKC, JAK/STAT, PKA/cAMP, retinoid, and PI3K/Akt pathway (**Table** **2** and **Figure** **3**B). In all the screening experiments, the base media + 0.1% DMSO was used as the control. Since the addition of PD032, Chir, and LIF (2i/LIF) maintains ES cells in a naïve pluripotent state and promotes their self‐renewal, this condition was included as a reference for differentiation.^[^ ^31^ ^]^ Structures were exposed to these modulators for the entire duration of the experiment (0 –120 h, Figure 3A), after which they were stained, imaged, and the numbers and ratios of XEn/EPiCs and other phenotypic classes were quantified in each condition using the CPA classifier module described above (Figure 2C).

**Table 2:** Library of signaling pathway modulators.

Modulators (in text abbreviation)	Pathway	Company	Concentration
CHIR‐99021 (Chir)	GSK3 inhibitor	Bio‐Techne Sales Corp. 4423/10	3 µm
XAV‐939 (XAV)	Tankyrase Inhibitor	StemCell Technologies 72674	4 µm
IWP‐2	Wnt/porcupine Inhibitor	StemCell Technologies 72124	2 µm
IM‐12	GSK‐3β inhibitor	Sigma‐Aldrich SML0084	1 µm
IWR‐1‐endo	Wnt/Catenin Inhibitor	StemCell Technologies 72562	2.5 µm
Rspondin	Wnt receptor Activator	R&D Systems 4645‐RS‐025	100 ng mL^−1^
SB431542 (SB43)	Tgfβ (ALK) Inhibitor	Abcam ab120163	10 µm
PD0325901 (PD032)	MEK/ERK Inhibitor	Bio‐Techne Sales corp 4192/10	1 µm
PD98059 (PD98)	Fgf/MAPK Inhibitor	SelleckChem S1177	10 µm
Fgf4 + heparin	Fgf/MAPK activator	R&D systems (Fgf4); Sigma (Heparin)	Fgf4‐25 ng mL^−1^; Hep‐ 1 µg mL^−1^
PD173074	FGFR Inhibitor	StemCell Technologies	0.1 µm
SU5402	FGFR Inhibitor	StemCell Technologies 73914	40 µm
Na Orthovanadate	TK inhibitor	Sigma S6508	40 µm
LDN‐193189 (LDN)	BMP inhibitor	R&D Systems 6053/10	600 nm
Dorsomorphin (Compound C) 2HCl (DM)	BMP inhibitor	SelleckChem S7306	0.5 µm
BMP4	BMP activator	R&D Systems 5020‐BP‐010	50 ng mL^−1^
Noggin	BMP Inhibitor	Stem Cell Technologies 78 061.1	300 ng mL^−1^
ML347	ALK1/2 inhibitor	Tocris 4945	1.5 µm
A‐83‐01 (A83)	Tgfβ/ALK5 Inhibitor	Tocris 2939	1 µm
LY364947	Tgfβ/Nodal inhibitor	SelleckChem S2805	20 µm
Activin A	Nodal/Activin activator	R&D systems 338‐AC‐050	50 ng mL^−1^
Tgfβ1	Tgfβ activator	Peprotech 100–21	2 ng mL^−1^
Nodal	Nodal activator	R&D systems 1315‐ND‐025	50 ng mL^−1^
RA/Tretinoin (NSC 122 758)	RA activator	Sigma‐Aldrich R2625	10 µm
UVI 3003	RXR inhibitor	Tocris 3303	10 µm
AGN 193109	RAR inhibitor	Tocris 5758	10 µm
LIF	JAK/STAT activator	Sigma‐Aldrich ESG1106	10 ng mL^−1^
SC‐144	JAK/STAT inhibitor	R&D systems 4963	0.5 µm
WP1066	JAK inhibitor	SellekChem S2796	5 µm
G1	Gp130 agonist	Tocris 3577	200 nm
Insulin	P13/Akt activator	Sigma‐Aldrich 91077C	50 ng mL^−1^
PT‐1	Ampk activator	R&D Systems 4039/10	50 µm
Y‐27632 2HCl	ROCK inhibitor	Enzo life sciences ALX‐270‐333‐M005	2 µm
Gö 6983	PKC inhibitor	MedChemExpress HY‐13689	2 µm
HA‐100 dihydrochloride	PKC inhibitor	SelleckChem S2964	10 µm
Indolactam V	PKC activator	MedChemExpress HY‐12307	10 µm
8‐Bromo‐cAMP	PKA activator	Biolog Life Science Institute BLG‐B007‐500	1 mm
H 89 2HCl	PKA inhibitor	SelleckChem S1582	10 µm
DL‐adrenaline	PKA activator	FisherScientific 15495279	100 µm

The pathway modulators were then grouped based on the proportion of identified phenotype classes (as described in Figure 2). The percentages mentioned here are relative to the total of five phenotypes (100%) in each condition. The modulators BMP4, activin A, Tgfβ1 (BMP/Tgfβ pathway agonists), and Rspondin (Wnt agonist) showed a XEn/EPiCs proportion of 68%, 64%, 70%, and 70%, respectively, which was slightly lower but not significant compared to the 77% found in the control (Figure 3C). This observation showed that the addition of these modulators from 0–120 h had minimal to no effect on the differentiation and organization of XEn, as well as on the efficiency of XEn/EPiCs formation. Interestingly, XAV (Wnt inhibitor), LDN and Noggin (BMP inhibitors), Fgf4 (Fgf activator), and Nodal (Nodal activator) showed an overall reduction of 40%, 55%, 57%, 57%, and 30% respectively, for the proportion of XEn/EPiCs (Figure 3C). Together, these observations show that activation of Tgfβ/Nodal and BMP signaling and amplified canonical Wnt signaling by Rspondin do not interfere with the XEn and Epi morphogenesis. However, their inhibition significantly affects the overall proportion of XEn/EPiCs.

The modulators Chir, SB431542 (SB43), PD98, A83, dorsomorphin (DM), WP1066, and DL‐adrenaline showed a significant reduction of XEn/EPiCs formation in lieu of a significantly higher proportion of XEn/Epi rosettes with an increase from 6% in the control to 9%, 18%, 20%, 16%, 21%, 37%, 25%, respectively (Figure 3C). In addition, there was a higher proportion of XEn/non‐polarized Epi in Chir (17%), SB43 (19%), PD98 (58%), DM (28%), and cAMP (39%) (Figure 3C). This observation is interesting because the above‐mentioned phenotypes represent an earlier stage of epiblast morphogenesis. This result suggests that these modulators either incited a roadblock on cavitation, thereby hindering its progression toward forming a PAC, or they slowed down developmental progression. Among these modulators, DM, cAMP, and WP1066 showed more dispersed XEn in comparison to the single epithelialized XEn in control, indicating the role of the BMP, PKA, and JAK/STAT pathways in XEn specification and patterning. Together, these results indicate that the inhibition of pathways such as, Tgfβ, BMP, Nodal, and JAK signaling, plays coordinated roles in the epithelialization of XEn, while Wnt signaling affects the timing of developmental progression to form PAC. It is important to note that Wnt activation and inhibitory timings are crucial during the pre‐ to post‐implantation transition and modulating Wnt by constant exposure to either Chir or XAV affected the overall formation of XEn/EPiCs.^[^ ^15^ ^]^

The inhibitors of the Fgf/MAPK pathway, specifically, PD032 (MEK/ERK inhibitor) and PD17 (FGF receptor inhibitor) resulted in 100% of EB‐like structures (Figure 3C), showing their failure to specify XEn cells. These findings are in line with the established role of Fgf signaling in the specification of XEn in the blastocyst, provided by inductions from the Epi.^[^ ^24^ ^]^ Signaling pathway modulators that showed a high proportion of amorphous XEn/Epi included LIF (100%), Indolactam (100%), LY36 (100%), IWR‐endo (100%), AGN (100%), UVI3003 (61%), RA (78%), and ML347 (84%) (Figure 3C). Together, this result shows that the prolonged exposure to these signaling pathway modulators affected the correct spatial organization and sorting of XEn lineage cells. Overall, this primary screen confirmed the role of BMP/Tgfβ, activin/Nodal, Fgf/MAPK, Wnt, and JAK/STAT signaling pathways in early embryo development.

### 2.4 Partitioning Signaling Pathway Modulation by Morphogenetic Time Windows

The specification and patterning (i.e., sorting out) of XEn and Epi in mouse blastocysts occur between 48 and 96 h after fertilization through the Grb2‐MAPK pathway,^[^ ^11b^ ^]^ which corresponds to the window of 0–72 h in the XEn/EPiC model. Exit from naïve pluripotency and establishment of a polarized post‐implantation epiblast epithelium occur in natural embryos between 96 and 120 h, which is followed by the formation and expansion of the pro‐amniotic cavity within 24 h.^[^ ^1d^ ^]^ This stage of development corresponds with the culture of XEn/EPiCs between 48 and 120 h. Morphometric analysis of XEn/EPiC cultures exposed to different pathway modulators at this time window could indicate their potential role in this process.

To increase the temporal resolution and gauge the effect of signaling pathways at specific chronological stages during the development of XEn/EPiCs, we chose a subset of modulators from the library to perform a secondary screen, Chir, XAV, Rspondin, Fgf4+Heparin, PD032, BMP4, DM, Tgfβ1, Nodal, activin A, SB43, and A83. These modulators either had minimal to no effect on the XEn/EPiCs or showed distinct phenotypes under the different classifications in the primary screen and significantly affected one or more of the morphological events such as XEn specification and patterning, Epi polarization, and/ PAC formation. The XEn/EPiCs were exposed to the chosen modulators for two distinct windows of development, namely 0–72 h and 48–120 h, to further delineate the role of these pathways in specific morphogenetic processes during development, and study the plasticity of the cells to catch up with the developmental program when perturbed.

**Figure 4:**
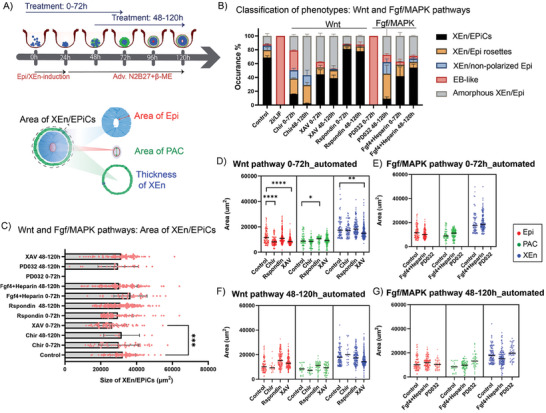
Automated phenotypic quantification of XEn/EPiCs exposed to signaling modulators of Wnt and Fgf/MAPK pathways: A) Top: experimental design of the exposure of XEn/EPiCs to different pathway modulators from 0–72 h and 48–120 h of development. Bottom: schematic showing the tissue compartment measurements on XEn/EPiCs, B) The effect of short‐listed signaling modulators of Wnt and Fgf/MAPK pathways on the yield of different structures at 0–72 h and 48–120 h, C) Effect of the modulators on the overall size of XEn/EPiCs, Area of Epi, PAC, and XEn in XEn/EPiCs when exposed to Wnt pathway modulators at D) 0–72 h, and F) 48–120 h, and Fgf/MAPK pathway modulators at E) 0–72 h, and G) 48–120 h; Control is base media + 0.1% DMSO (0–72, 48–120 h). Data are mean ± s.d. obtained from *n* = 3 wells, with each well containing ≈150 structures. After filtering for only XEn/EPiCs, sample size depended on the ratio of phenotypes observed in the overall population. In the graphs, each dot represents one XEn/EPiC. All statistical hypothesis testing was done using Dunnett's test; ∗ represents *P <*= 0.05, ∗∗ represents *P* < 0.01, ∗∗∗ represents *P* < 0.001, ∗∗∗∗ represents *P* < 0.0001 (One‐way ANOVA with Dunnet's post‐test).

**Figure 5:**
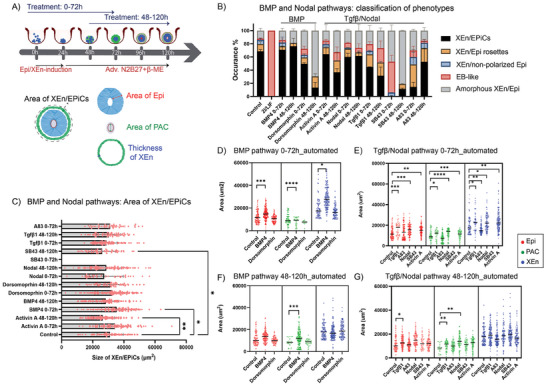
Automated phenotypic quantification of structures exposed to signaling modulators of BMP and Tgfβ/Nodal pathway: A) Top: experimental design of exposing the pathway modulators from 0–72 h and 48h–120 h of development of XEn/EPiCs. Bottom: schematic showing the tissue compartment features measured on XEn/EPiCs, B) The effect of short‐listed signaling modulators of BMP and Tgfβ/Nodal pathways on the yield of different structures at 0–72 h and 48–120 h, C) Effect of the modulators on the overall size of XEn/EPiCs, Area of Epi, PAC, and XEn in XEn/EPiCs when exposed to BMP pathway modulators at D) 0–72 h, and F) 48–120 h, and Tgfβ/Nodal pathway modulators at E) 0–72 h, and G) 48–120 h; Control is base media + 0.1% DMSO (0–72, 48–120 h). Data are mean ± s.d. obtained from *n* = 3 wells, with each well containing ≈150 structures. After filtering for only XEn/EPiCs, sample size depended on the ratio of phenotypes observed in the overall population. In the graphs, each dot represents one XEn/EPiC. All statistical hypothesis testing was done using Dunnett's test; ∗ represents *P<* = 0.05, ∗∗ represents *P* < 0.01, ∗∗∗ represents *P* < 0.001, ∗∗∗∗ represents *P* < 0.0001 (One‐way ANOVA with Dunnet's post‐test).

The effect of activators and inhibitors of different pathways on the XEn/EPiCs at 0–72 h and 48–120 h are schematically shown in **Figures** **4**A and **5**A. In addition to quantifying the ratio of different phenotypes, the data obtained from CP were fed‐back into the CPA classifier module to systematically filter XEn/EPiCs from the images and measure the area of individual tissue compartments (Figures S6–S10, Supporting Information). This method of quantifying individual cellular compartments in an automated set‐up is important because it is informative to identify the effect of different pathways on the developmental progression of XEn/EPiCs and to determine how much these pathways are involved in regulating tissue morphogenesis.

#### 2.4.1 Wnt Pathway

##### Wnt Activation Affected the Formation of PAC While its Inhibition Affected XEn Patterning with a Reduction in the Size of XEn/EPiCs

From the primary screen, it was observed that modulation of the Wnt pathway with Chir, XAV, or Rspondin affected different aspects of XEn/EPiCs development. To observe and systematically quantify the effect of this pathway on Epi differentiation and polarization, XEn/EPiCs were exposed to signaling pathway modulators for two time windows. The exposure of structures to Chir from 0–72 h showed a higher number of XEn/Epi rosettes (23% vs 10% in control) than XEn/EPiCs (15% vs 70% in control) (Figure 4B) with 30% of the structures resembling an EB‐like phenotype (Figure 4B). Similarly, exposure from 48–120 h showed a higher occurrence of XEn/Epi rosettes (26% vs 9% in control) and amorphous XEn/Epi (60% vs 10% in control) and a low occurrence of XEn/EPiCs (4% vs 70% in control) (Figure 4B). Quantification of the overall size of Chir‐treated XEn/EPiCs showed no significant change in comparison to the control (Figure 4C).

By further analyzing the areas of the different compartments in the XEn/EPiCs, namely the Epi size, PAC area, and XEn thickness, in 0–72 h Chir treatment, we found a significant decrease in the size of Epi from 11000 µm^2^ in control to 10000 µm^2^ (Figure 4D). However, the size of PAC and XEn showed no significant effect upon Chir treatment (Figure 4D,F). Overall, the higher occurrence of structures presumably resembling an earlier time‐point of the embryo, namely XEn/Epi rosettes, XEn/non‐polarized Epi, and EB‐like structures, taken together, could indicate that there could be a slight delay in the developmental progression of XEn/EPiCs after Chir exposure from 0–72 h as well as from 0– 48 h.

The inhibition of Wnt with XAV treatment from 0–72 h, showed a reduced occurrence (42%) of XEn/EPiCs and a higher proportion of amorphous XEn/Epi (38% vs 10% in control) (Figure 4B) with a significantly reduced overall size of XEn/EpiCs (28000 µm^2^ vs 32000 µm^2^ in the control; *P <* 0.001; Figure 4C), likely contributed by the significant reduction in the size of Epi (10000 µm^2^ vs 11000 µm^2^ in the control; *P* < 0.0001) and XEn (17000 µm^2^ vs 20000 µm^2^ in the control; *P* < 0.01) (Figure 4D). This observation was also validated by manual quantification of the cellular compartments (Figure S12, Supporting Information). The 48–120 h exposure to XAV showed a similar occurrence of XEn/EPiCs, however, there was no significant effect on the size of different compartments, albeit with a dispersed XEn layer (Figure 4F). This observation suggests that prolonged Wnt inhibition by XAV from 0–72 h altered the timing of the developmental progression of these structures causing a smaller phenotype, while its exposure from 48–120 h affected the epithelialization of XEn.

Rspondins, co‐activators of Wnt, have been identified to increase the visibility of Wnt receptors and indirectly activate the Wnt pathway.^[^ ^32^ ^]^ In this screen, Rspondins showed a similar yield of XEn/EPiCs with 81% at 0–72 h compared to the control (70%). The sizes of individual compartments showed a similar ratio as in the control, although the size of PAC showed a marginal increase (11000 µm^2^ vs 10000 µm^2^ in the control) (Figure 4D). A similar effect on PAC was observed with 48–120 h treatment (Figure 4F). However, it was interesting to observe an increase, albeit not significant, in the Epi size from 10000 µm^2^ in control to 14000 µm^2^. This increase could have been due to endogenous Wnt signaling potentiation through Rspo1, thereby increasing Epi size via reinforcing a Wnt negative feedback loop.^[^ ^33c^ ^]^ Together, the analysis of the effect of Wnt pathway modulators validated the findings from the literature that show the effect of Wnt in XEn maintenance and sorting by regulating the catenin‐cadherin interactions via a feedback loop.^[^ ^33^ ^]^ Interestingly, the above data also showed that the inhibition of Wnt affected the timeline of the developmental progression of XEn/EPiCs thereby delaying the formation of an epithelialized XEn and expanded PAC.

#### 2.4.2 Fgf/MAPK Pathway

##### Activation of the Fgf/MAPK Pathway Reduced the Occurrence of XEn/EPiCs While its Inhibition from 0–72 h Showed an EB‐Like Phenotype

The Fgf/MAPK pathway has been widely studied for its key role in the specification and reinforcement of the XEn lineage in naive pluripotent cells.^[^ ^24^ ^]^ In addition, maintenance of the post‐implantation Epi cells relies on Fgf proteins,^[^ ^34^ ^]^ which imputes a rapid molecular switch in response to these signals. The exposure of ES cells to MEK inhibitor PD032 or FGF receptor inhibitor PD17 from 0–72 h led to the complete failure of structures to specify XEn (Figure 4B), which is similar to the phenotype observed with Fgf/MAPK signaling inhibition in mouse blastocysts.^[^ ^24^ ^]^ Interestingly, treatment with PD032 after the specification of XEn, from 48–120 h, allowed XEn maintenance but led to a drastically reduced yield of XEn/EPiCs (10%). This observation could indicate that prolonged Fgf inhibition at the later stage of development presumably slows down the transition of primed epiblast rosette into XEn/EPiCs, thereby resulting in a higher occurrence of XEn/Epi rosettes (35%) upon treatment with PD032 (Figure 4B). Hyperactivating Fgf/MAPK by the exposure to Fgf4 + Heparin showed 60% and 50% of structures forming XEn/EPiCs, at 0–72 h and 48–120 h windows, respectively. This observation suggests a larger sensitivity to Fgf/MAPK dosing in pre‐ than in peri‐implantation development (Figure 4B).

Quantification of the overall size of XEn/EPiCs in these conditions did not show a significant difference when compared to the control. A comparison of the area of Epi, PAC, and XEn in the XEn/EPiCs also did not show a significant difference compared to the control in both the time windows (Figure 4G). The algorithm did not perform area measurements in the case of treatment with PD032 and PD17 for 0–72 h because the majority of structures showed an EB‐like phenotype without XEn cells (Figure 4E). Overall, the Fgf/MAPK pathway inhibition from 0–72 h displayed a higher occurrence of EB‐like structures than the treatment from 48–120 h, thus emphasizing the 0–72 h crucial window for the specification of XEn.^[^ ^24^ ^]^

#### 2.4.3 BMP pathway

##### Activation of BMP Promoted the Developmental Progression of XEn/EPiCs and its Inhibition Affected the Sorting and Epithelialization of XEn

BMP signaling plays an important role in the establishment and sorting of XEn.^[^ ^12a^ ^]^ Exposure of ES cells to BMP4, which binds to the type‐1 receptors activating BMP signaling, from 0–72 h showed a slightly higher percentage (75%) of structures forming XEn/EPiCs to that of the control (70%) (Figure 5B). In contrast, treatment with BMP inhibitor 0.5 µm dorsomorphin (DM), a Type‐1 receptor inhibitor (Figure 5B) reduced the yield of XEn/EPiCs (25% vs 10% in control) with a significant ratio of structures representing the amorphous XEn/Epi structures with dispersed XEn. A similar effect was observed in the 48–120 h treatment.

Quantification of the sizes of individual tissue compartments in XEn/EPiCs upon treatment with BMP4 showed that there was a significant increase in the sizes of Epi (15000 µm^2^), and PAC (12000 µm^2^) compared to control (11000 and 10000 µm^2^ respectively) (Figure 5D). BMP4 inhibition with DM at both time windows of treatment showed comparable sizes of Epi, PAC, and XEn as the control (Figure 5D,F). Interestingly, the XEn in 0–72 h and 48–120 h XEn/EPiCs showed a more dispersed organization upon DM treatment (Figure 5D,F), a phenotype also observed in the primary screen (0–120 h) (Figure 3F). Together, the observations in both the time windows of exposure indicated that suppression of BMP signaling affected XEn sorting, aligning with the literature^[^ ^35^ ^]^ showing similar effects. The marginally increased sizes of Epi, XEn, and PAC in the BMP4 and DM‐treated structures should be further evaluated to validate the findings.

#### 2.4.4 Tgfβ/Nodal pathway

##### Tgfβ/Nodal Pathway Activation did not Show a Significant Effect on the Formation of XEn/EPiCs, Although XEn Sizes Showed Large Variability Upon their Inhibition

Nodal signals, through a self‐perpetuating loop between the Epi and XEn, are involved in patterning the visceral endoderm.^[^ ^36^, ^37^ ^]^ Here, the treatment of ES cells with Nodal pathway activators, activin A, and nodal, from 0–72 h had minimal impact on the yield of XEn/EPiCs with 68% and 65%, respectively, but was reduced to 45% and 60%, respectively, in 48–120 h treatment, in comparison to the control (70%) (Figure 5B). This was also seen with the reduced size of XEn/EPiCs (31400 µm^2^ vs 32000 µm^2^ in the control) in the 48–120 h treatment with activin A (Figure 5C). The more pronounced activity of activin A may be related to its independence of co‐factors Cripto/Cryptic to activate the receptor, in contrast to nodal.^[^ ^38^ ^]^ One unique observation was that the activation of Tgfβ/Nodal pathway with tgfβ1 from 0–72 h and 48–120 h, both showed a steep reduction in the yields of XEn/EPiCs (50% and 35% respectively) compared to 70% in the control. However, blocking the nodal and tgfβ pathway with SB43 and A83, respectively, at both time windows, drastically reduced the yield of XEn/EPiCs and displayed a higher yield of amorphous XEn/Epi and XEn/Epi rosettes, respectively (Figure 5B). This suggests a role for activin/nodal signaling in XEn establishment, in agreement with findings in ES cells that can be differentiated to XEn through a combination of activin‐A and Wnt activation.^[^ ^39^ ^]^

The overall size of XEn/EPiCs upon nodal treatment from 0–72 h was slightly but significantly reduced by 400 µm^2^ (*P* < 0.05) in comparison to the control (32000 µm^2^) (Figure 5C). On the contrary, inhibition of Tgfb/Nodal signaling by SB43 also led to a reduction in the size of XEn/EPiCs (27500 µm^2^ vs 32000 µm^2^ in the control, Figure 5C), which is in line with findings in Nodal^−/−^ embryos that are of smaller size.^[^ ^37^ ^]^ Together, this suggests a balanced Nodal activity as a requirement for size regulation, in agreement with its graded activity in regulating target genes.^[^ ^40^ ^]^ There was no significant difference in the size of XEn/EPiCs in other conditions. Quantification of the sizes of individual tissue compartments in XEn/EPiCs treated with tgfβ1, nodal, and activin A from 0–72 h, showed an increase in Epi with 19000, 17000, and 17000 µm^2^, PAC with 14000, 14500, and 13000 µm^2^, and XEn with 25000, 24500, and 25000 µm^2^, respectively, compared to the control (11000, 10000, 20000 µm^2^, respectively) (Figure 5E). The treatment from 48–120 h also showed a similar increasing trend in the sizes of Epi and PAC (Figure 5G). Despite indicating no significant change in the sizes of Epi, PAC, and XEn in SB43 and A83 treatment, the XEn layer was highly dispersed (Figure 5G). Together, these findings indicate the important role of Tgfβ/Nodal signaling in XEn patterning and PAC formation where inhibition led to a drastic reduction in XEn/EpiCs formation efficiency.

## 3 Discussion

Automated detection and quantification holds high potential for the future since it provides an efficient means to streamline the focus on finding the most relevant and interesting phenotypes in 3D cell structures like embryo models. High‐content phenotypic screening of suspension‐based 3D complex cell structures, such as some organoids and embryo models,^[^ ^9^, ^18^, ^19^, ^41^ ^]^ requires a versatile platform that simultaneously allows reproducible and long‐term culture, and in situ staining and image‐based readouts. The vast amount of image data should be efficiently and (semi‐)automatically analyzed and be interpretable for cell biologists. Therefore, we chose a machine‐learning‐assisted approach to classify the multiple embryo‐like phenotypes in our cultures using unbiased feature measurements. Then, in the phenotype of interest, we developed an additional automated analysis pipeline to identify and measure the relevant tissue compartments. The advantages of our analysis approach are that it is performed in a user‐friendly, open‐source software environment, requiring easy drag and drop‐based supervised machine learning, allows multiple phenotypic classifications in the same algorithm, and finally allows exporting the rules generated for each phenotypic class back into CP^[^ ^21^ ^]^ to enable filtering and morphometric read‐outs.

The role of different signaling pathways in the specification and patterning of extraembryonic endoderm (XEn) as well as in the polarization and expansion of the epiblast have been well studied in the literature.^[^ ^12^, ^35^ ^]^ However, it is not very clear how different pathways individually exert their influence on this stage of development and to what extent they affect different tissue types, especially in the case of pro‐amniotic cavity (PAC) formation and expansion. In this screen, for the first time, we performed a qualitative (image‐based) and quantitative (machine‐learning assisted) analysis on the XEn, Epi, and PAC layers in the embryo‐like structures. Additionally, we also observed that modulation of specific pathways such as Wnt and Fgf/MAPK at specific time windows emphasized the importance of time of activation/inhibition during the pre‐ to post‐implantation morphogenesis in embryo‐like structures.

The primary screen exposing the embryo‐like structures to pathway modulators for the entire duration of development, 0–120 h, showed some interesting effects, with some of the activators and inhibitors of the same pathway displaying similar effect on XEn/EPiCs upon treatment. This effect could be observed because the embryo‐like structures go through multiple developmental time windows during this period interfering with the stages where either agonistic or antagonistic signals of that pathway are required. Hence, despite observing similar phenotypic ratios of structures, the compounds might have exerted their effect at different time points within that window. This effect was more pronounced in Wnt and Fgf/MAPK pathway modulators, Chir/XAV and Fgf4/PD98 respectively.

In the case of BMP pathway modulators, differential inhibitory effects were observed when the embryo‐like structures were exposed to different BMP inhibitors, namely noggin, dorsomorphin (DM) and LDN‐193189 (LDN). DM produced a more pronounced effect on the yield of XEn/EPiC formation in comparison to noggin and LDN, which showed similar phenotypic effects. Target specificity could be one explanation; noggin primarily inhibits various BMP types,^[^ ^12d^ ^]^ whereas both DM and its derivative LDN act as inhibitors of both SMAD (downstream of BMP) and non‐SMAD pathways. Additionally, DM has been shown to produce significant “off‐target effects” against AMP‐activated protein kinase (AMPK), and vascular endothelial growth factor type‐II receptor (VEGFR‐II).^[^ ^12c^ ^]^ The effects of LDN beyond BMP cannot be ruled out as potential contributors to the effect observed. However, the observed effect is similar to that of noggin. A second reason could be the differential effect of noggin and DM on the development of the extraembryonic endoderm (XEn) and trophectoderm (TE). According to,^[^ ^12c^ ^]^ in E4.5 mouse embryos, 1 µm DM treatment led to a more severe reduction in the number of PrE (XEn) and TE cells (91% and 54% respectively), in comparison to 300 ng mL^−1^ noggin. This result could indicate a similar mechanism occurring in the DM and noggin treated XEn/EPiCs, whereby DM showed a more pronounced effect on the yield of XEn/EPiCs and patterning of XEn in our screen. This differential effect between the BMP pathway inhibitors should be explored further to understand the mechanism of action of these BMP inhibitors.

**Figure 6:**
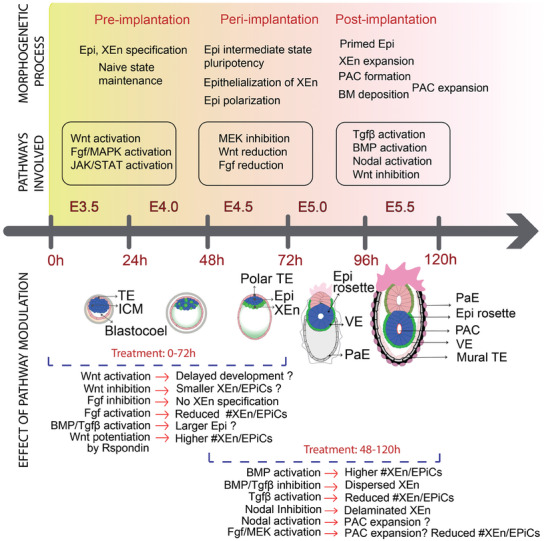
Schematic summary of the timeline of mouse embryogenesis displaying the morphogenetic processes and signaling pathways involved in each stage of development. In this screen, different pathway modulators were used to delineate and quantitatively study the effect of signaling pathways on the development of mouse pre‐ to post‐implantation embryo‐like structures; Legend: Epi‐epiblast, XEn‐ extraembryonic endoderm, TE‐ Trophectoderm, ICM‐ inner cell mass, PAC‐ pro‐amniotic cavity, VE‐ visceral endoderm, PaE‐parietal endoderm, BM‐ basement membrane.

To delineate the effect of specific pathway agonist and antagonist, the structures were exposed to pathway modulators for two different time windows of development. Exposure to BMP4 from 48–120 h showed a higher yield of formation of XEn/EPiCs, which could be attributed to the role of BMP signaling in XEn expansion,^[^ ^12^, ^35^ ^]^ and may have also contributed to the PAC formation. Meanwhile, the exposure to BMP pathway inhibitors (DM, noggin) from 0–72 h as well as 48–120 h, and Tgfβ/Nodal inhibitors (SB43, A83) from 0–72 h, affected the XEn specification, patterning, and PAC formation. It is known that nodal signaling provides instructive cues for the transition of XEn to visceral endoderm (VE)^[^ ^3c^ ^]^ and supports β‐integrin‐mediated bonding between the epithelialized XEn and Epi^[^ ^27^ ^]^ via basement membrane (BM) production,^[^ ^37^ ^]^ thereby allowing the PAC to expand (**Figure** **6**). Similarly, exposure to Fgf/MAPK inhibitors (PD032, PD17) from 0–72 h, during which the Epi/XEn specification occurs in XEn/EPiCs, completely interrupted the differentiation of XEn, leading to an EB‐like phenotype. This observation is in alignment with previous reports indicating the role of the BMP, Fgf/MAPK, and Tgfβ/Nodal pathways on the specification and patterning of extra‐embryonic lineages in mouse embryos.^[^ ^12^, ^24^, ^42^ ^]^ Further, it opens up the discussion on how incorrect XEn organization restricts the formation and expansion of the PAC. Some interesting findings from the analysis pipeline were the effects of Rspondin (0–72 h), Tgfβ1 (0–72 h), BMP4 (48–120 h), nodal (0–72 h, 48–120 h), and SB43 (48–120 h), which displayed a significant increase in Epi thickness. These findings should be further explored for their biology, for example, whether the Epi has an enriched pseudo‐stratified alignment of cells, as in vivo embryos,^[^ ^1^, ^43^ ^]^ and if the Epi cells change shape or size with the exposure to some factors.

Wnt has been reported to play a multitude of roles in development depending on the spatiotemporal expression and activator‐inhibitory patterns.^[^ ^44^ ^]^ During the 0–72 h window, the Epi transitions toward a state of rosette‐stage pluripotency and becomes a polarized epithelium.^[^ ^15^ ^]^ Activating the Wnt pathway using Chir led to reduced yields of XEn/EpiCs and instead higher yields of XEn/Epi rosettes and XEn/non‐polarized Epi. This could indicate a delay in the formation of the PAC, possibly due to the blockage of the naive to rosette‐stage epiblast transition, which is necessary for cavity formation^[^ ^15^ ^]^(Figure 6). One of the speculations is that continued Wnt activation, prolongs the naïve pluripotency, and hence, showed a reduced yield of XEn/EPiCs as well as a reduced size of the epiblast. Wnt activation sustains pluripotency in mouse ES cells and subsequent Wnt inhibition is required for the pluripotency transition into that of a formative stage epiblast during the post‐implantation progression.^[^ ^15^ ^]^ One of the interesting observations was that the inhibition of Wnt by XAV from 0–72 h or 48–120 h resulted in smaller structures with relatively smaller Epi and sometimes showed a dispersed organization of XEn. It has been shown that Wnt supports XEn expansion^[^ ^15^ ^]^ and so, prolonged Wnt inhibition may have affected the developmental progression of these structures, causing a higher proportion of amorphous XEn/Epi. Another speculation is that there is a minimum cell number required for the formative stage epiblast to proceed development of the PAC, which, when blocked by the inhibition of Wnt, led to a premature differentiation and thereby, smaller XEn/EPiCs. Another unique finding was that the exposure to Rspondin at all time windows (0–120 h, 0–72 h and 48–120 h) increased the yield of XEn/EPiCs and also showed an expansion of Epi at 48–120 h exposure. This observation could be possibly related to an increased endogenous Wnt signaling potentiation, causing a Wnt negative feedback loop.^[^ ^33c^ ^]^

A limitation of the automated readout is that in some of the structures the size of the XEn layer was measured inaccurately due to its occasional delamination from the Epi, possibly a result of the absence of trophoblast signals in this model.^[^ ^45^ ^]^ Further inspection showed that this phenotype was affected by specific modulators in the screen, which needs further validation using volumetric imaging of the structures. To dissect the delaminated from non‐delaminated XEn, we could then perform a secondary machine‐learning training class to differentiate between delaminated and non‐delaminated embryo‐like structures and further filter out the desired phenotype to perform other morphometric measurements.

In summary, Wnt, Fgf/MAPK, BMP, and Tgfβ /Nodal pathways together were found to play a significant role in the pre‐ to the post‐implantation progression of XEn/EPiCs as well as in the development of distinct embryonic tissue types. Overall, the culture of embryo‐like structures in our imaging platform and the quantification of different features of the structures using CP and CPA showed considerable overlap with literature findings on embryo development. Importantly, the screens on embryo‐like structures provided new hypotheses (Figure 6) on the molecular underpinnings of embryo morphogenesis that may be tested in other mouse embryo models. Moreover, other embryo models with increased complexity, like ETX embryos,^[^ ^3a,b^ ^]^ can expectedly also be translated to our novel platform. In conclusion, we provide a culture and screening platform that is highly scalable and versatile, which can be applied to different kinds of screening experiments, such as testing the toxicity of drugs, testing the teratogenicity of factors, and identifying disease phenotypes.

## 4 Experimental Section

### Mouse ES Cell Line

The Gata6‐h2b‐Venus ES cell line was a kind gift from C. Schröters’ laboratory (Max Planck Institute of Molecular Physiology, Dortmund, Germany.^[^ ^46^ ^]^ All the experiments were conducted using these cells within passages 10–18. The ES cell line was tested for mycoplasma.

### Formation and Culture of XEn/EPiC Structures within Thermoformed Microwells

*Preparation of Thermoformed Microwells*: STATARRAYs (Polystyrene microwell 96‐well plate from 300MICRONS GmbH) was used for the 3D culture of mESCs.^[^ ^20^, ^47^ ^]^ Before usage, the wells were washed 1x with 70% ethanol and 2x with water. The wells were then incubated with an Anti‐adherence solution (StemCell Technologies) for ≈20 min at RT inside the laminar flow hood and then washed 3x with PBS and then incubated with fresh PBS until further use.

*ES Cell Culture and Reagents*: Mouse embryonic stem cells (mESCs) were expanded on a feeder layer of mouse embryonic fibroblasts (MEFs) on 0.15% gelatin in ES medium containing, Dulbecco's Modified Eagle's Medium High Glucose (Life Technologies) supplemented with 10% fetal bovine serum (FBS, Greiner), 4 mm Glutamax (Life Technologies), 100 U mL^−1^ penicillin (Life Technologies), 100 mg mL^−1^ streptomycin (Life Technologies), 10 mm non‐essential amino acids (Life Technologies), and freshly supplemented with 0.1 mm 2‐mercaptoethanol (Life Technologies), 1000 U mL^−1^ leukemia inhibitory factor (LIF, Life Technologies), 3 µm CHIR99021 (GSK3β inhibitor, Axon Medchem) and 1 µm PD0325901 (MEK/ERK inhibitor, Sigma Aldrich). The cells were refreshed every 2 days, passaged on the 3rd day with 0.5 mL Accutase for 3 min, and seeded at a density of 10000 cells cm^−2^ along with 0.5 µm Y27632. For all the experiments in this article, mES:: Gata6: H2B: Venus reporter line was used (mESCs comprising an H2B‐Venus reporter under the regulatory elements of Gata6).

*Culture of XEn/EPiCs*: XEn‐induction medium containing advanced N2B27 medium was prepared as follows: 46.3% Advanced DMEM/F12 (Invitrogen), 46.3% Neurobasal (Invitrogen), 1% N2 supplement (Invitrogen), 2% B27 supplement (Invitrogen), 1% Glutamax, 1% Non‐Essential Amino Acids, 1.5% BSA (Sigma), 0.5% HEPES, 0.4% Sodium Pyruvate), 3 µm CHIR99021, 0.1 mm 2‐mercaptoethanol, 1 mm 8Br‐cAMP, 25ug mL^−1^ Fgf4, 1 ug mL^−1^ Heparin, 10 nm Retinoic acid, 1 µm Y27632, 5% FBS, and 1% Pen/Strep. 25 µL of the medium was added to all the wells and placed inside the incubator.

mESCs were washed 2x with PBS to remove the dead cells, treated with 0.5 mL Accutase for 3 min, centrifuged at 200 g for 5 min, and the pellet was suspended in 7 mL MEFs medium containing DMEM (high glucose, Sodium pyruvate, and Glutamax) with 15% FBS. MEFs depletion involved seeding the cell suspension first onto a non‐coated T75 flask, to allow MEFs to adhere to the plate, for 20–30 min. The cell suspension was then collected from the flask without mixing, centrifuged again, and the pellet re‐suspended in 1 mL of adv. N2B27 medium. After counting, the cell suspension was made with XEn‐ind medium at a concentration of 160000 cells mL^−1^, and 50 µL of it was added to a tube containing 150 uL of the XEn‐ind medium. Finally, 200 µL of cell suspension was added to each well and placed back in the incubator for the cells to settle.

In all the screening experiments, the basal medium control was advanced N2B27 medium supplemented with 100 µm β‐Mercaptoethanol and 1% Pen/Strep. The first 24 h of culture had an addition of XEn induction factors that were added to the medium. After 24 h, the induction factors were washed off and the basal medium (advanced N2B27 medium with 100 µm β‐Mercaptoethanol and 1% Pen/Strep) was continued until the end of culture (120 h), with refreshing medium every 24 h. For the treatment conditions, the respective compounds were added on top of the existing basal media, and for the control 0.1% DMSO was added to the basal media at 0–72 h and 48–120 h. 2i/LIF was added as a reference to show the undifferentiated, pluripotent EB‐like population.

*Fixation and Staining*: At 120 h, the structures were washed 3x with PBS and fixed with a fixing solution containing ice‐cold 2% PFA and 0.1% Glutaraldehyde for 30 min at RT. The wells were then washed 3x with PBS and continued for further staining or stored at 4 ^ο^C.

Staining was performed by permeabilizing the structures with 0.1% Triton‐x 100 or 1% Tween‐20 for 30 min. at RT. Then, the wells were treated with Hoechst 33 342 (1:300), Phalloidin AF647 (1:300), and WGA CF568 (1:300) in 0.1% Triton‐x 100 and incubated for 30 min. at RT. The wells were then washed 3x with PBS and stored at 4 °C or imaged under the microscope.

*Imaging*: The structures were imaged using an automated inverted Nikon Ti‐E live cell spinning disk confocal imaging microscope, equipped with environmental control and a CrestOptics X‐Light V2 spinning disk unit with a pinhole size of 40 µm or 70 µm. For all automated measurements a 20X air objective was used (CLWD (O.D. = 2.10 mm, NA = 0.5)). To perform manual measurements of the area of individual compartments (Figure S12, Supporting Information) a 40X air‐immersion objective (O.D. = 0.66 mm, NA = 0.75) was used. The “large image” module was used to achieve a montage of the entire well with all the microwells stitched together. Manual image analysis was performed using NIS software and ImageJ (yield and area measurement). All the images were taken using epi‐fluorescence imaging in a single focal plane, roughly at the plane where the size of the cavity is at maximum. This is based on the assumption that most structures are spherical and the cavity forms in the center plane of the structures.

### CellProfiler Pipeline Creation and Setup

*Measurement of Features*: The analysis of 3D structures was performed using CellProfiler (CP) v4.1.3 and CellProfiler Analyst (CPA) v3.0.4 (Broad Institute). Each structure within a montage image of a well was identified as an individual object and each object was then quantified for its gross feature measurements such as area, size, shape, texture, intensity, and intensity distribution including Zernicke features using the modules “MeasureObjectArea”, “MeasureObjectIntensity”, “MeasureObjectIntensityDistribution”, “MeasureObjectSizeShape”, and “MeasureTexture”. The obtained whole‐structure measurements were then exported into a database file for use with CPA and a spreadsheet for further analysis (Figure S1, Supporting Information). All files and pipeline can be shared upon request.

*Supervised Training*: For supervised training of the classification algorithm, the different structures (or objects) in each well were manually classified into 5 classes based on visual morphological parameters such as the presence of a polarized, rosette‐shaped Epi, specification of Gata6+ cells, epithelialization of Gata6+ cells, and presence of a pro‐amniotic cavity in the center of the Epi. Using these visual parameters, the different objects were manually dragged into different classes to create a training set. The size of the training sets in the experiments was in the range of 25–30 structures per class. A training set was established by scoring and evaluating the training using “Fast‐gentle boosting” algorithm of machine learning, which produces specific rules for each class using the gross feature measurements obtained from CP. After the supervised training, the training set was evaluated for accuracy, precision, recall, and F1 score, which were 85%, 84%, 76%, and 80% respectively for XEn/EPiCs (Figures S2,S3, Supporting Information).

*Yield Quantification using CPA*: The output from the CP pipeline was imported into CPA and the classifier module was used to perform a supervised machine‐learning algorithm based on the “fast‐gentle boosting” scoring method to classify the different phenotypes of objects observed. The algorithm uses the measurements obtained from CP to create a set of rules for each class. To use the same training set for different rounds of experimentation, there were more structures added to the training sets to check the accuracy of prediction. Once the training set had been established, it was used to “score” the whole dataset (all the treatment conditions and controls with replicates, roughly 288 wells/experiment). This module gives the score of the yield of different classifications of structures based on gross feature measurements.

There was an additional “others” class, where the experimental artifacts and out‐of‐focus images were sorted out. Most images only had a few structures in the “others” class (<2%). The CPA software was then trained to generate a set of rules based on the measurements from CP (Figure S1, Supporting Information). After a satisfactory training set, the images were scored based on the number of objects falling into each class.

*Quantification of the Area using CP*: The rules generated after the training with classifier module were imported back into the CP pipeline under the “Filter objects” module and the objects falling into each class could be filtered out in each image (Figures S1,S6, Supporting Information). Further morphometric measurements were performed on the resulting objects by identifying the individual tissue compartments and measuring the ratios of areas of Epi, XEn, and PAC as explained in Section 4.4.

### Identification and Quantification of Individual Compartments within Structures

*Area of Epiblast (Epi) with Cavity*: The area of Epi with pro‐amniotic cavity was obtained by using the “Image Math” module to subtract the Gata6 channel from the Hoechst channel (Figures S4,S8,S9, Supporting Information).

*Area of Pro‐Amniotic Cavity (PAC)*: The PAC compartment was obtained by using the “Image Math” module to multiply the area of the Epi with cavity with F‐Actin channel (Figures S4,S10, Supporting Information).

*Area of Extraembryonic Endoderm (XEn) Layer*: The area of XEn was determined by subtracting the area of Epi with cavity from the overall area of the Hoechst channel (Figures S4,S11, Supporting Information).

### Statistical Analysis

All data obtained from CP consisting of yield ratios were pre‐processed by converting the different ratios of the phenotypes observed into percentage values per experiment using Microsoft Excel. All the area measurements obtained from the CP were directly imported into GraphPad Prism (v10.0.2.232) for further analysis. All data were presented as mean ± SD. All experiments were conducted in triplicates with each treatment condition generating upto 169 structures per replicate. The sample size (n) for yield measurements were ≈160 structures. For area measurements, the XEn/EPiCs were filtered out based on their ratio within the whole population and were measured for all replicates (*n* = 3). The sample size for statistical analysis of area measurements could be in the range of 80–120 structures depending on the treatment condition. In the graphs, each dot represents one XEn/EPiC. All statistical hypothesis testing was done using Dunnett's test; ∗ represents *P*< = 0.05, ∗∗ represents *P* < 0.01, ∗∗∗ represents *P* < 0.001, ∗∗∗∗ represents *P* < 0.0001 (One‐way ANOVA with Dunnet's post‐test).

## Conflict of Interest

S.G. is one of the founders and a shareholder of 300MICRONS GmbH.

## Supporting information

Supporting Information

## Data Availability

The data that support the findings of this study are available from the corresponding author upon reasonable request.
