# Supplementary material for: Automated, High‐Throughput Phenotypic Screening and Analysis Platform to Study Pre‐ and Post‐Implantation Morphogenesis in Stem Cell‐Derived Embryo‐Like Structures
Source: Adv Sci (Weinh). 2023 Nov 22;11(4):2304987. doi: 10.1002/advs.202304987 (PMC10811479; doi:10.1002/advs.202304987)
Supplement: Supplementary file 1 — Supporting Information [file ADVS-11-2304987-s001.pdf]

ADVANCED SCIENCE
Open Access

Supporting Information

for *Adv. Sci.*, DOI 10.1002/advs.202304987

# Automated, High-Throughput Phenotypic Screening and Analysis Platform to Study Pre- and Post-Implantation Morphogenesis in Stem Cell-Derived Embryo-Like Structures

*Vinidhra Shankar, Clemens van Blitterswijk, Erik Vrij* and Stefan Giselbrecht**

## Supporting information

Flow chart of the pipeline for CP and CPA to identify and measure different parameters

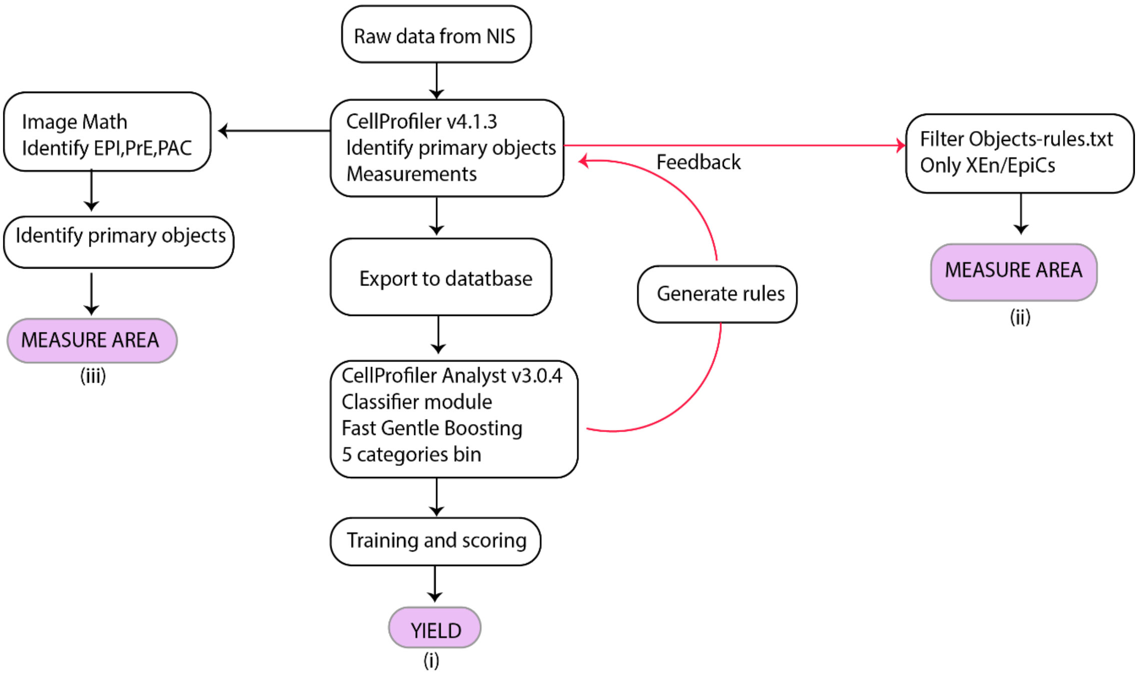


***Figure S 1: Flowchart of the pipeline for CP and CPA to identify and measure different phenotypic parameters.***

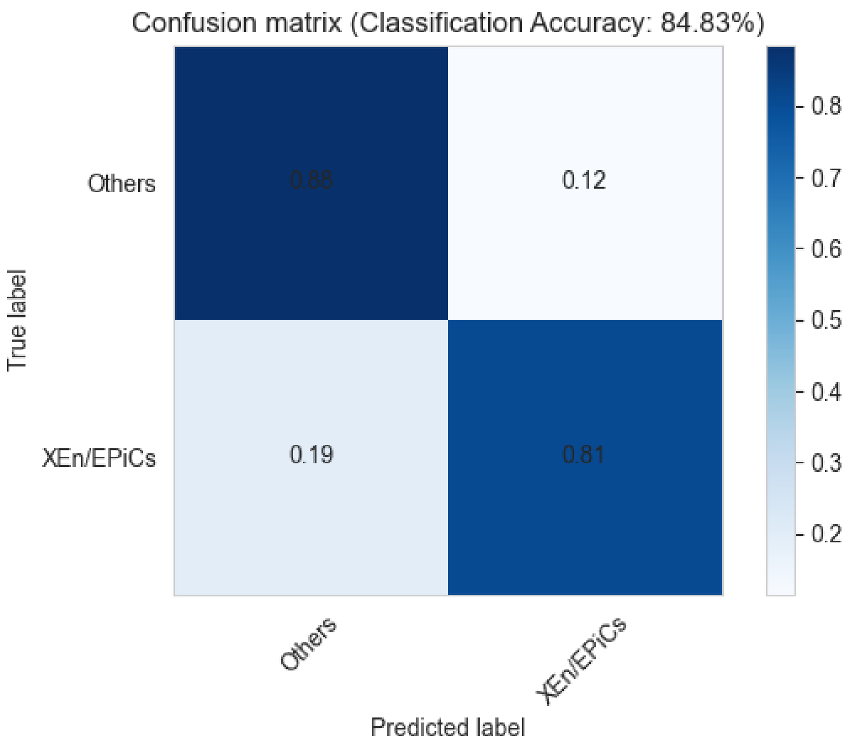


***Figure S 2: % Accuracy of the automated, supervised machine-learning algorithm for predicting how well the system detects XEn/EPiCs, the phenotype of interest, in comparison to other phenotypes; top left: true negative, top right: false negative, bottom left: false positive, bottom right: true positive***

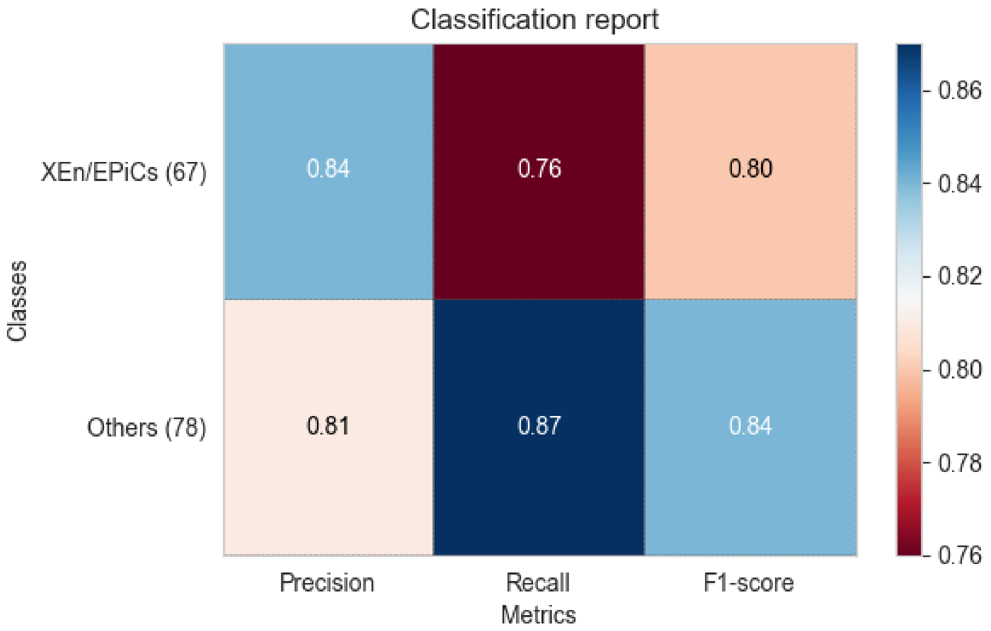

***Figure S 3: Precision, recall and F1 score for evaluating the performance of the machine learning algorithm used in this screen. The scores are compared for how well the system detects XEn/EPiCs, the phenotype of interest, in comparison to other phenotypes.***

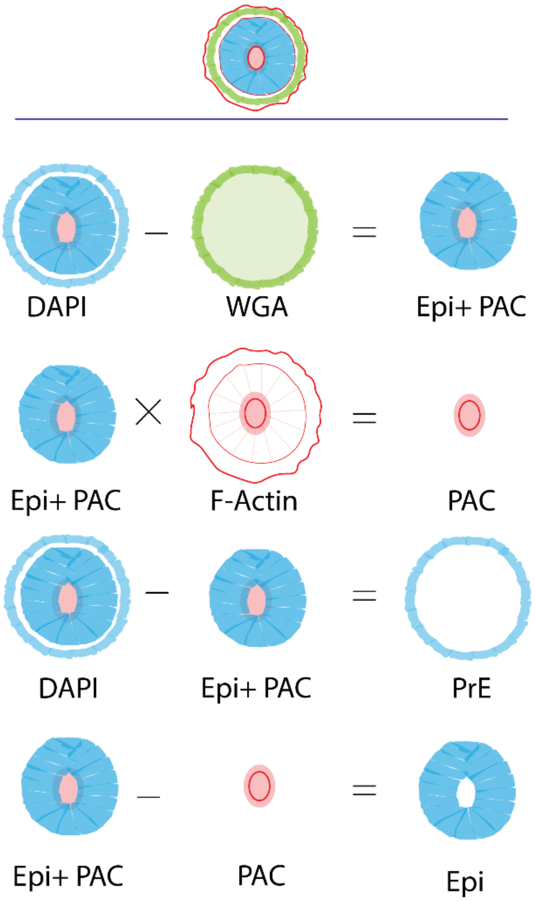


***Figure S 4: ImageMath feature to identify individual tissue compartments within XEn/EPiCs***

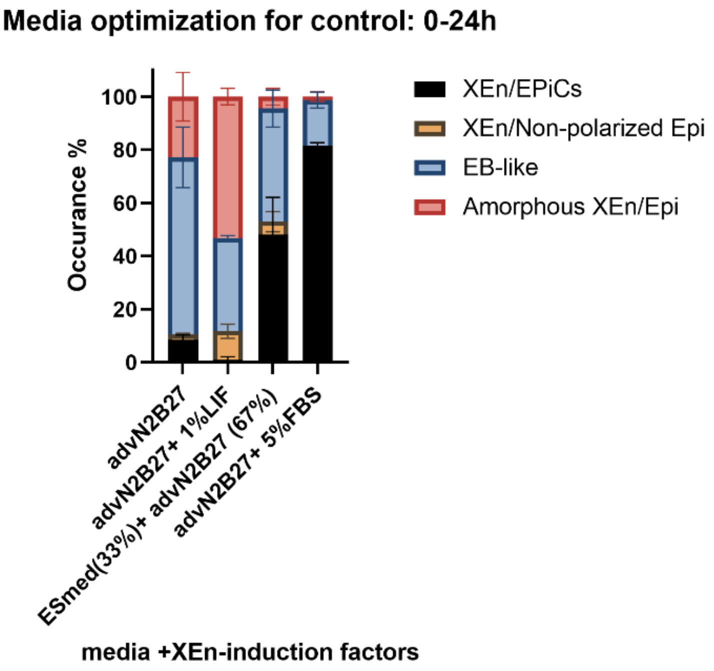


***Figure S 5:Media optimization for the formation of XEn/EPiCs within thermoformed microwells:*** mESCs were seeded into thermoformed microwells with (i) advanced N2B27 media, (ii) advanced N2B27 media + 1% LIF, (iii) 33% ESmed+ 67% adv.N2B27, (iv) advN2B27+5% FBS. All the media conditions were tested with XEn-induction factors from 0-24h of ESCs seeding. After that, all conditions were refreshed with advanced N2B27 + 0.2% β-mercaptoethanol + 1% Penicillin/Streptomycin until 120h. The media condition with advanced N2B27 + 5% FBS-based XEn-induction media gave the highest percentage of XEn/EPiCs with 80%.

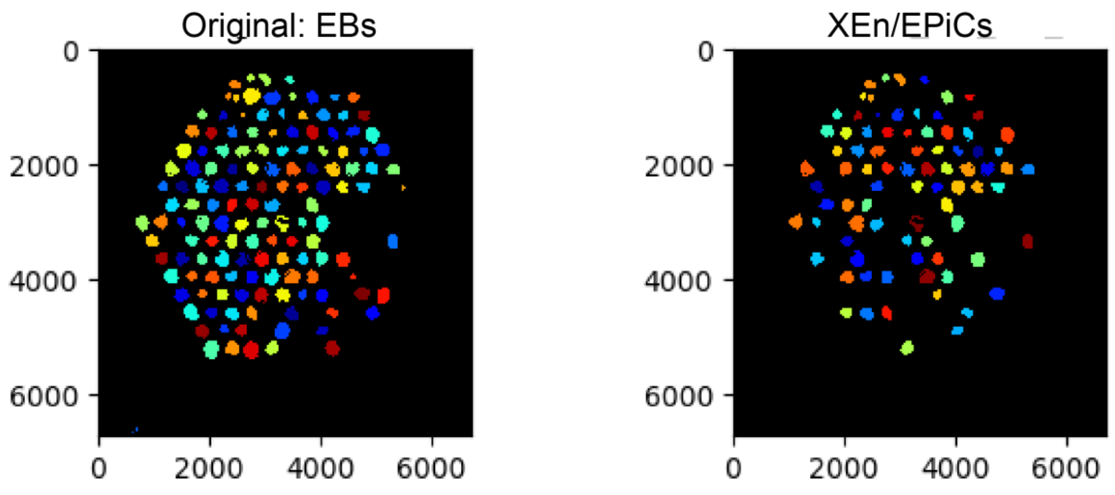


| | |
|---|---|
| Number of objects pre-filtering | 140 |
| Number of objects post-filtering | 82 |
| Number of objects removed | 58 |

***Figure S 6: CellProfiler module runs for Filter objects command***: The 'Filter objects' module is feedback from the rules generated from CellProfiler Analyst that filters the objects for XEn/EPiCs only.

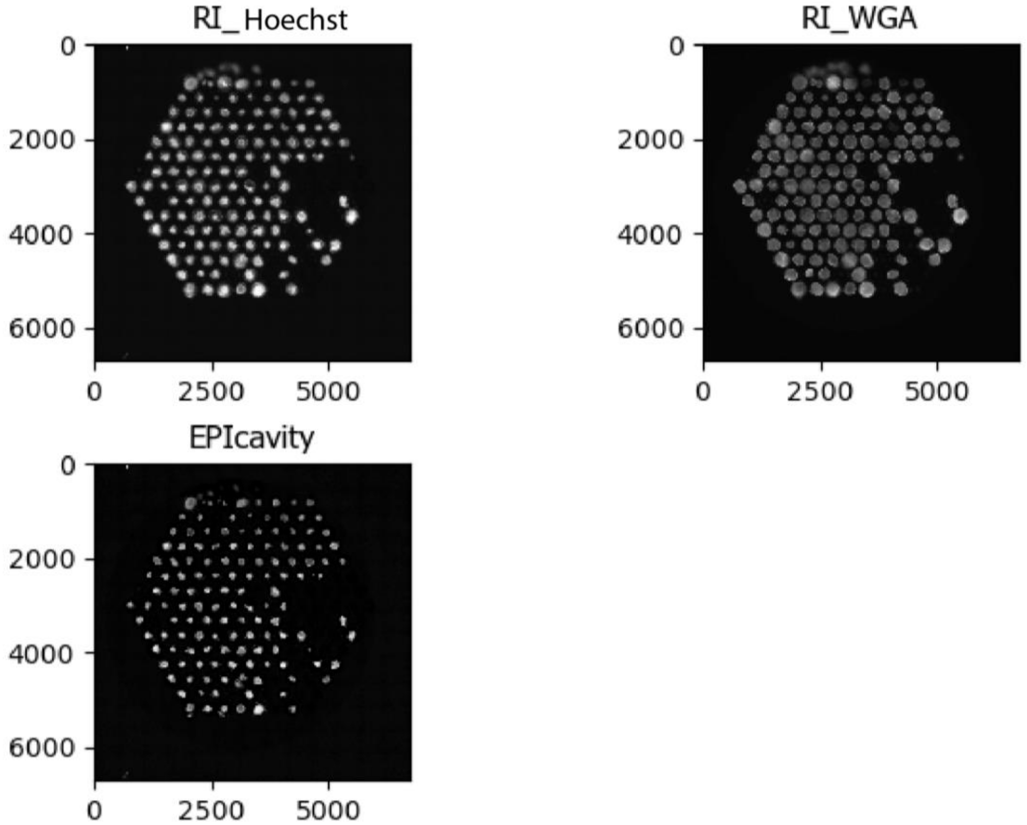


***Figure S 7: ImageMath for identifying Epi+cavity:*** The 'ImageMath' module performs the subtraction of RescaleIntensity of Hoechst (RI_Hoechst) and RescaleIntensity of WGA (RI_WGA) to give the resulting image of Epiblast + pro-amniotic cavity (EPIcavity).

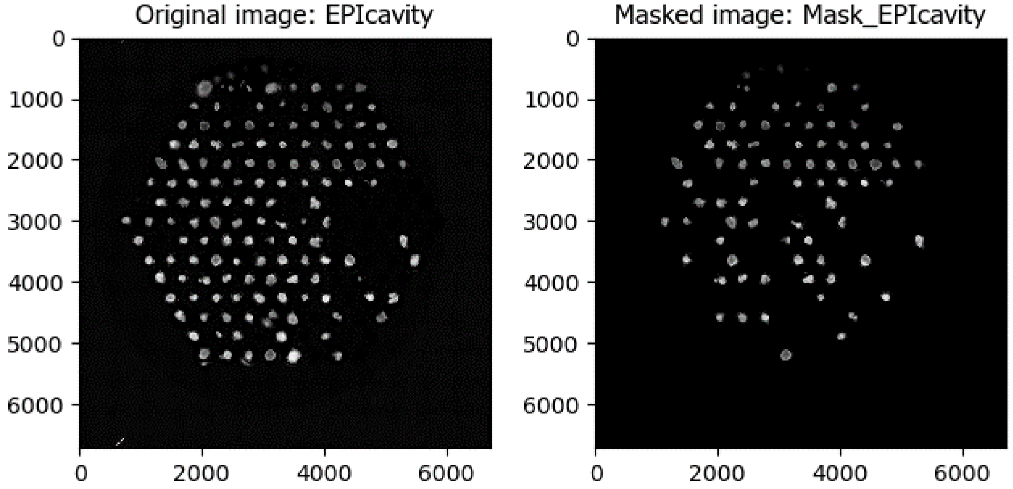


***Figure S 8: Masking of EPIcavity with Filterobjects****: 'MaskImage' module creates a mask of XEn/EPiCs from the Filterobjects module to show only the EPIcavity within XEn/EPiCs.*

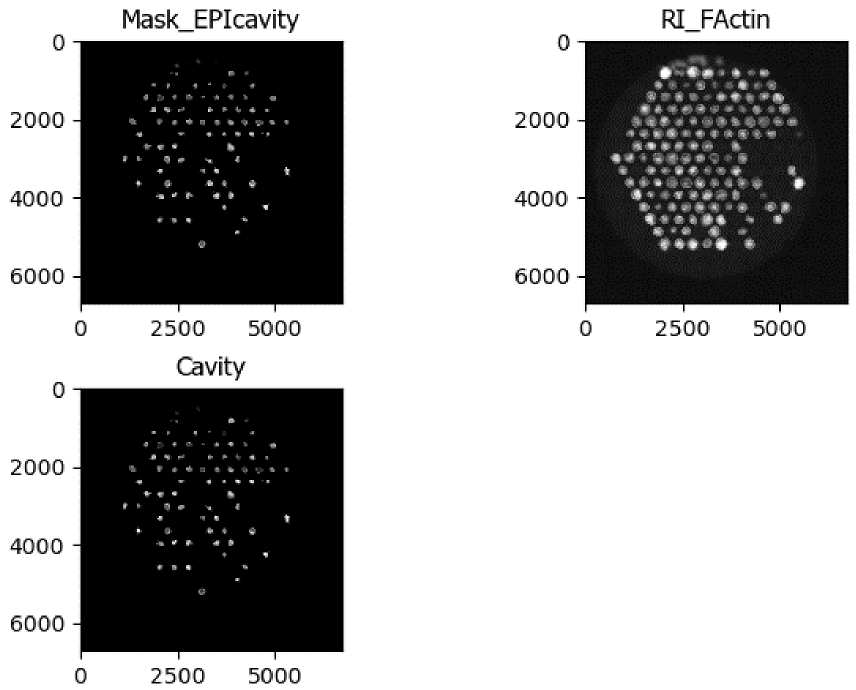


***Figure S 9: ImageMath for identifying Cavity:*** The 'ImageMath' module performs the multiplication of masked images of EPIcavity (Mask_EPIcavity) and RescaleIntensity of F-Actin (RI_FActin) to give the resulting image of the pro-amniotic cavity (Cavity)

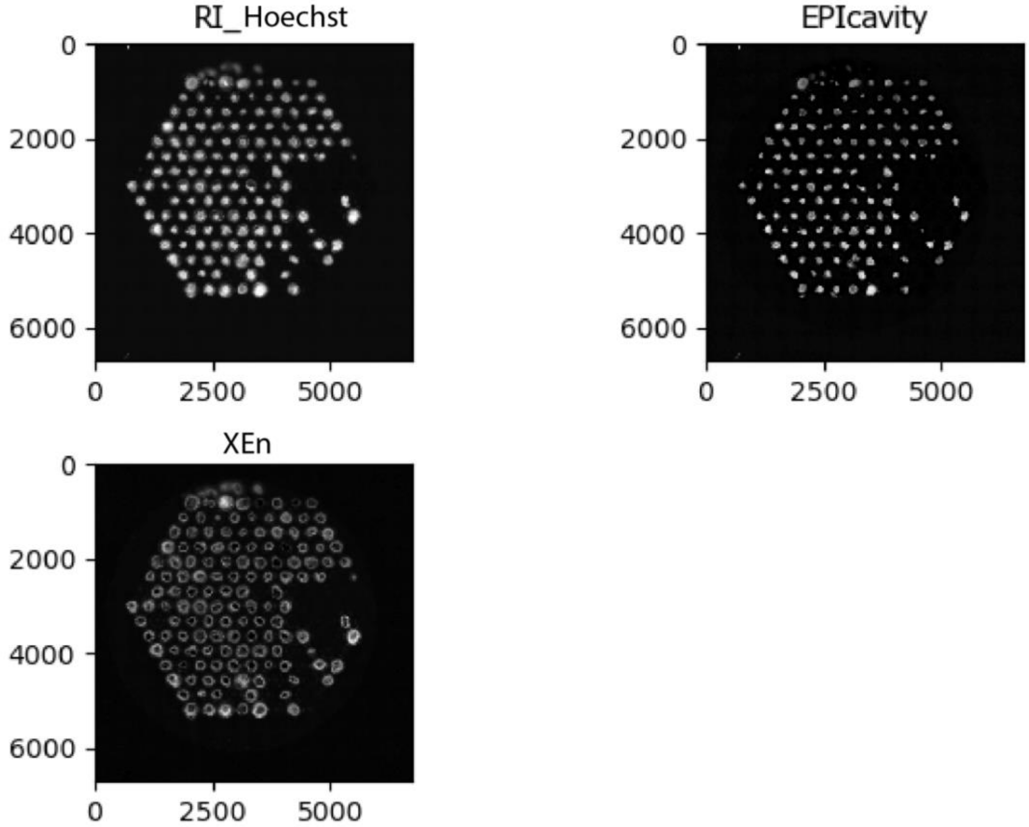


***Figure S 10: ImageMath for identifying XEn:*** The ‘ImageMath’ module performs the subtraction of masked images of RescaleIntensity of Hoechst (RI_Hoechst) and epiblast + PAC (EPIcavity) to give the resulting image of the XEn

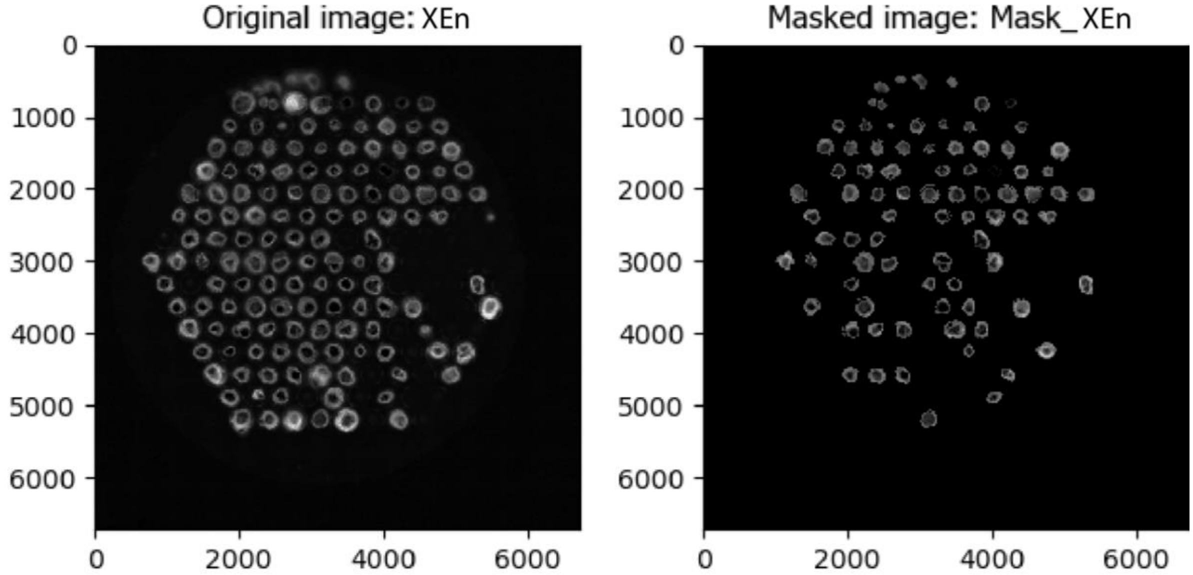


***Figure S 11: Masking of XEn with XEn/EPiCs:*** *‘MaskImage’ module creates a mask of XEn/EPiCs from the Filterobjects module to show only the XEn within XEn/EPiCs*

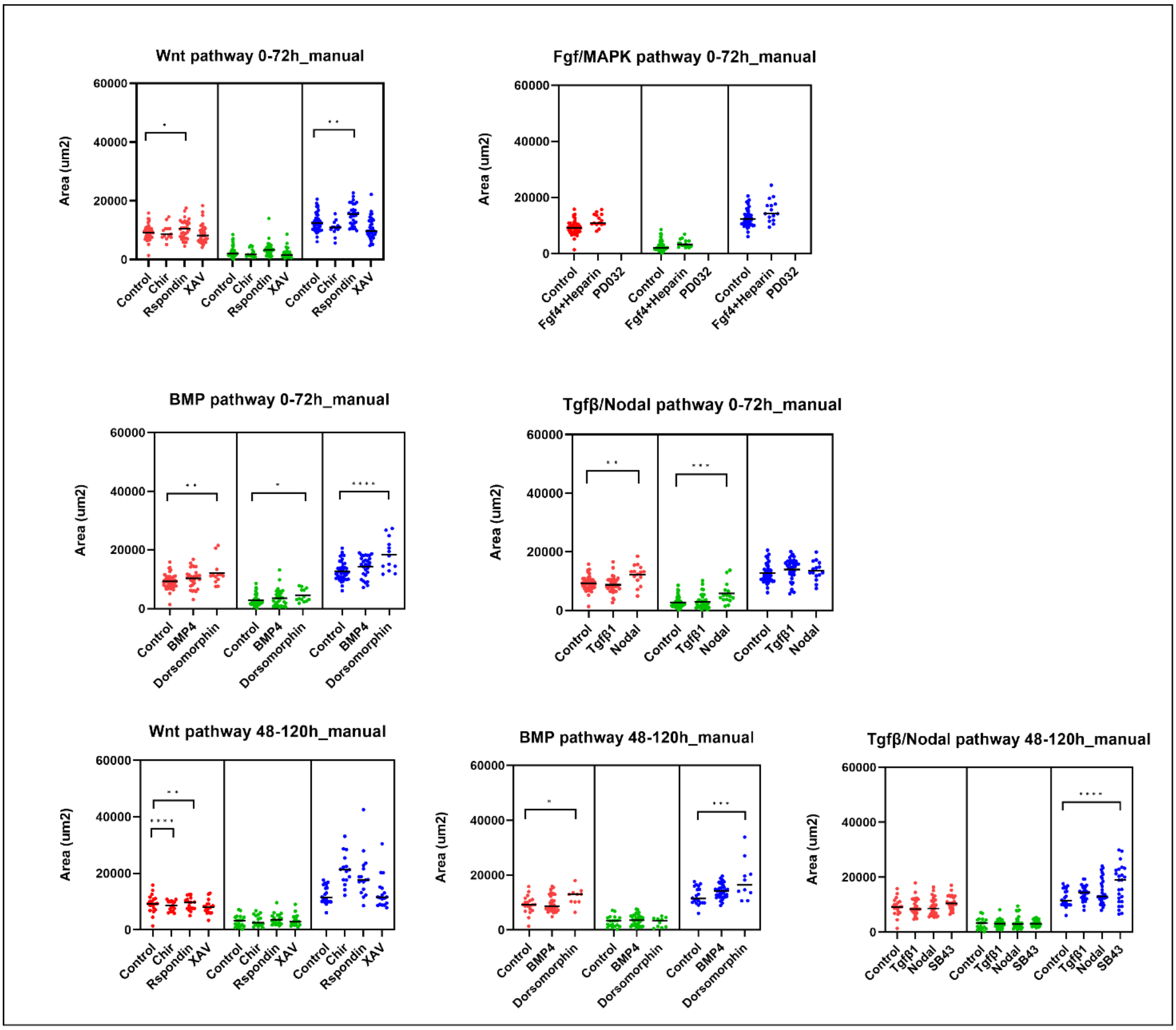


*Figure S 12: Manual measurements of the Epi, PAC and XEn compartments of 0–72 h and 48–120 h XEn/EPiCs*
